# Neurobiochemical Effects of a High-Fat Diet: Implications for the Pathogenesis of Neurodegenerative Diseases

**DOI:** 10.3390/biology14101317

**Published:** 2025-09-24

**Authors:** Marta Srokowska, Wojciech Żwierełło, Agata Wszołek, Izabela Gutowska

**Affiliations:** 1 Department of Medical Chemistry, Pomeranian Medical University in Szczecin, Powstańców Wlkp. 71 St., 70-111 Szczecin, Poland; m.srokowska02@gmail.com (M.S.); izabela.gutowska@pum.edu.pl (I.G.); 2 Institute of Biology, University of Szczecin, Felczaka 3c St., 70-453 Szczecin, Poland; agata.wszolek@usz.edu.pl

**Keywords:** brain metabolism, cognitive impairment, high-fat diet, hippocampus, ketogenic diet, neurodegeneration, neuroinflammation, oxidative stress, synaptic plasticity

## Abstract

The global rise in high-fat diet (HFD) consumption and obesity has raised concerns about their long-term effects on brain health. This review addresses how HFDs, including ketogenic diets (KDs), influence the central nervous system (CNS) and may contribute to neurodegenerative processes. The findings show that prolonged HFD exposure is associated with altered brain metabolism, increased oxidative stress, neuroinflammation, and impaired synaptic plasticity, particularly in regions like the hippocampus and hypothalamus. These changes may affect cognitive function and accelerate neurodegenerative mechanisms linked to disorders such as Alzheimer’s and Parkinson’s disease. While certain types of KD appear to exert neuroprotective effects—such as improved motor outcomes in experimental Parkinson’s disease models—evidence remains inconsistent, and concerns about their long-term safety persist. This review emphasizes that the impact of high-fat nutrition on the CNS depends on fat type, exposure duration, and individual factors such as age and sex. Overall, further research is needed to distinguish between harmful and potentially therapeutic dietary fat patterns and to better understand their influence on brain health across the lifespan.

## Simple Summary

This article discusses how a high-fat diet affects brain function and the development of neurodegenerative diseases. The goal was to summarize current knowledge on how excessive fat consumption—especially in the form of ketogenic diets—impacts brain metabolism, inflammation, and cognitive function. The authors show that such diets can cause oxidative stress, memory decline, and disruptions in key brain areas like the hippocampus, hypothalamus, and dopamine system, and even affect brain development in offspring. However, ketogenic diets may offer protective effects in some cases, particularly in Parkinson’s, Alzheimer’s, or ALS, although their long-term safety is still uncertain. Most studies cited are based on animal models, which limits their direct applicability to humans. The authors conclude that caution is needed with high-fat diets and that further research is essential to distinguish between harmful and potentially beneficial fats and dietary patterns. This topic has significant societal value, especially given the popularity of weight-loss diets.

## 1. Introduction

Nutrition significantly influences proper physiological function. Each person requires a specific amount of energy for an active, healthy lifestyle, as well as appropriate proportions and types of nutrients necessary for normal growth, development, and tissue regeneration [1]. Currently, an increasingly common global issue is overweight and obesity among adults and children. It is estimated that, in 2020, obesity affected 158 million children and adolescents aged 5–19 years, with projections of 206 million by 2025 and 254 million by 2030. This trend represents a major public health problem due to its contribution to other metabolic diseases such as cardiovascular disease and diabetes [2]. The primary cause of obesity is generally considered an imbalance between energy intake and expenditure. When the body receives more energy than it can use, excess carbohydrates are stored as glycogen in muscles and liver or as triacylglycerols in adipose tissue. Environmental factors, including dietary habits and the type of diet, play a crucial role in this balance. Among these, a high-fat diet is regarded as a significant contributor to obesity [3]. The expanding “pandemic” of overweight and obesity is driven by a steady rise in global production and consumption of fats and oils of plants (mainly palm, soybean, sunflower, and rapeseed oils) and animal origin. These fats, which vary greatly in their fatty acid profiles, are nutrient-dense and widely available [4]. It is currently very important to limit the intake of saturated fatty acids (SFA) and replace them with unsaturated fatty acids (UFA). The composition of these plant oils—characterized by lower SFA and higher UFA content—has a more beneficial effect on the body. Reports suggest that greater dietary intake of UFA is associated with a lower risk of type 2 diabetes. Moreover, higher consumption of essential plant-derived polyunsaturated fatty acids reduces atherogenic lipids and lipoproteins, thereby decreasing the risk of heart disease [5]. In contrast, SFA linked to an increased risk of cardiovascular diseases and metabolic disorders such as obesity (overweight) and type 2 diabetes, mainly due to their association with elevated blood cholesterol [6]. Preclinical studies reviewed here have demonstrated that both short-term and long-term consumption of HFD leads to biochemical changes and behavioral alterations in the organism [5].

A variant of the HFD is the KD, whose growing popularity is linked to its promising use in treating headaches and neurodegenerative diseases. However, many individuals adopt KD for weight loss, body-shaping, or lifestyle changes. Concerns remain about its long-term safety: some studies report beneficial effects of KD, while others document negative outcomes. This diet has sparked controversy due to divergent perceptions of its benefits—enthusiasm among certain patient and weight-loss communities versus skepticism from parts of the medical community [7].

In both clinical and non-clinical practice, several variants of the ketogenic diet (KD) are used:

(1) Classical KD (4:1 or 3:1 ratio): The ratio refers to grams of fat to the combined grams of protein and carbohydrates, typically providing 70–90% of energy from fat. This variant is especially applied in drug-resistant epilepsy [8].

(2) Medium-chain triglyceride KD (MCT-KD): A portion of dietary fat is supplied as medium-chain triglycerides, which allows for higher protein and carbohydrate intake while maintaining ketosis. Clinical reviews indicate that its efficacy is comparable to the classical KD [9].

(3) Modified Atkins Diet: Characterized by more liberal protein intake and carbohydrate restriction to typically 10–20 g/day, without the need for weighing meals. It is often used in adolescents and adults [9].

(4) Low Glycemic Index Treatment (LGIT): Involves maintaining a glycemic index <50, with moderate carbohydrate restriction and a higher proportion of dietary fat [10].

Additionally, exogenous ketones (β-hydroxybutyrate salts and esters) are used to temporarily increase ketonemia without strict carbohydrate restriction [11]. Athletes are increasingly turning to ketone supplements to enhance ketone body availability without adhering to a restrictive diet. Commercially available ketone supplements include medium-chain triglycerides (MCT), β-hydroxybutyrate salts and monoesters with 1,3-butanediol, as well as acetoacetate mono- and diesters with 1,3-butanediol (AcAc) [12,13]. The aim of this review is to present current research findings on the role of HFD in central nervous system function and the prevention of neurodegenerative disorders.

## 2. Materials and Methods

Peer-reviewed scientific articles were analyzed from the PubMed and Google Scholar databases, using relevant keywords and phrases: “high-fat diet”, “HFD”, “brain”, “ketogenic diet”, “KD”, “ketone bodies”, “ketogenesis”, “fatty acids”, “CNS” “hippocampus”, “microglia”, “hypothalamus”, “dopaminergic system”, “offspring”, “neurodegenerative diseases”, “Alzheimer’s disease”, “Parkinson’s disease”, “Huntington disease”.

Also, combinations of these phrases were used, such as:

“((High fat diet) OR (HFD)) AND (brain)”;

“((Ketogenic diet) OR (KD)) AND (neurodegenerative diseases)”;

“(HFD) AND (hippocampus)”, “(HFD) AND (microglia)”.

The selection criteria focused on recent studies addressing high-fat or ketogenic diets and their effects on neurobiology and neurodegeneration. The review covered the years 2000–2025 (searches last updated on 16 September 2025; databases: Pub-Med/Medline, Scopus, Web of Science, Google Scholar). Older publications were included only if they represented seminal or foundational works.

## 3. High-Fat Diet and Body Metabolism

The brain requires an adequate energy supply for its basic metabolic activity. Nutrients are delivered across the blood–brain barrier (BBB) because the brain’s own energy reserves cover only a fraction of its total demand. In conditions of limited glucose availability—or during periods of intensive growth—the brain can utilize alternative energy sources such as KB or lactate [14]. In adults, the brain accounts for approximately 2% of body mass but consumes nearly 20% of resting energy expenditure (BMR/RMR), highlighting the high energetic cost of neuronal function [15]. In children, the brain represents a much larger proportion of total body mass, reaching up to 50%. The total rate of cerebral metabolism corresponds to about 20 W, or ~0.25 kcal/min. Assuming that this energy is used primarily for the synthesis of high-energy phosphate bonds, with an efficiency of ~20% [16]. KB generates more ATP per molecule than glucose, enabling efficient energy production even under calorie restriction [17].

A high-fat diet is a nutritional strategy characterized by increased fat intake combined with a strong reduction in carbohydrates (typically to about 50 g/day), while maintaining adequate protein intake. The main goal of such a diet is to shift energy metabolism toward fat utilization and elevate circulating ketone concentrations to promote weight loss [18]. This diet contains large amounts of fatty acids but is often low in fiber, vitamins, and minerals [19]. In a classical HFD, fats constitute 40–60% of total energy, carbohydrates 20–40%, and protein 10–20%. Scientific studies have associated this type of diet with the development of insulin resistance, obesity, diabetes, and neurodegenerative diseases [20]. It has also been linked to certain cancers of the gastrointestinal tract (e.g., colorectal cancer), primarily via so-called “metabolic reprogramming” [19]. Among fatty acids, SFA are key drivers of chronic metabolic inflammation, whereas unsaturated fatty acids (both monounsaturated and polyunsaturated) exert anti-inflammatory effects and help maintain energy homeostasis [21].

Earlier diets with similar nutritional principles include the Atkins diet (without protein restriction), the South Beach diet, and the Zone diet [22]. Today, the ketogenic diet has become the most popular HFD variant. KD’s fundamental premise is to keep carbohydrate intake very low while varying fat and protein levels. A classic KD typically provides 1 g of protein per kg of body weight, 10–15 g of carbohydrates per day, and the remaining caloric needs from fats [23]. Historically, this dietary approach was used to treat epilepsy (a practice noted as far back as the New Testament). Other variants of KD include the MCT diet, the low glycemic index treatment, and the modified Atkins diet [24]. In most cases, classical KD can only be used for a short time, as it requires consuming an extremely large proportion of fats (sometimes up to 90% of calories). Consequently, many practitioners favor the MCT diet, in which the main fats consumed are capric and caprylic acids [25].

KD aims to increase production of ketone bodies (β-hydroxybutyrate, acetoacetate, and acetone) in the liver. This diet is meant to mimic a fasting state without the negative effects of actual starvation. Adherence to KD increases fatty acid oxidation and promotes the utilization of the resulting ketone bodies as an alternative energy source [26].

Ketosis is a natural metabolic state in which blood ketone levels rise from about 0.2 mM to over 3.0 mM. It can be induced physiologically by physical activity, fasting, or starvation, and can also occur pathologically, for example, in the course of diabetes [27]. The use of low-carbohydrate, high-fat diets leads to increased production of KB, which serve as an alternative energy source under conditions of limited glucose availability (glucose being the brain’s main energy source) [28]. Such a metabolic state is called nutritional ketosis and persists as long as the body is deprived of carbohydrates, with gluconeogenesis and ketogenesis being the primary metabolic pathways involved [29]. Restricting carbohydrate intake leads to decreased insulin levels in the blood and, as a result, to reduced activity of the lipogenesis pathway. The long-chain fatty acids present in typical dietary fats are transported in combination with proteins (lipoproteins), which prevents them from crossing the BBB and being used as an energy source by the brain. When glycogen stores are insufficient to meet the demands of the CNS for glucose and to support fat oxidation, the body requires an alternative energy source, which becomes ketone bodies synthesized in the liver through ketogenesis. Under carbohydrate restriction, acetoacetate accumulates, which is then converted to acetone and βHB [30]. The effect of this metabolic state is an increase in blood ketone concentration and their appearance in urine and exhaled air [31]. Acetone serves as a marker in exhaled breath (BrAce) and is used to assess fat metabolism (ketone body levels) [32]. Its measurement is particularly important for the diagnosis and monitoring of diabetes, especially type 1. In healthy individuals, BrAce concentrations typically range from 0.3 to 1.0 ppm, whereas in patients with type 1 diabetes they may be higher, particularly during ketosis, exceeding 1.7 ppm [33]. Some publications suggest that HFD causes impaired glucose tolerance. In a study on young mice, it was shown that this diet caused increased hyperglycemia, which may indicate the development of an early prediabetic state characterized by dysregulation of blood glucose levels without insulin resistance. In addition, an increased level of leptin was found in plasma, most likely due to increased adipose tissue mass [34].

## 4. Inflammation in the Nervous System as a Possible Consequence of HFD

Inflammation is a biological process that constitutes the first line of defense of the organism upon exposure to harmful factors. Its most common sources include bacterial and viral infections, allergen exposure, radiation, harmful chemicals, chronic and autoimmune diseases, as well as obesity and high-calorie diets [35,36]. The inflammatory response can follow two main pathways. Acute inflammation is short-term (lasting from a few minutes to several days) and nonspecific; it is characterized by plasma protein or fluid leakage and the migration of leukocytes into the extravascular space. Chronic inflammation, in contrast, is long-lasting (persisting for months or years) and specific; it most commonly develops as a result of infections that are not eliminated by host defense mechanisms or other forms of immunity [37].

Adipose tissue in obese individuals releases free fatty acids (FFA) during triglyceride degradation. FFA and lipopolysaccharides bind to Toll-like receptor 4 (TLR4) on macrophages, initiating the production of inflammatory mediators. The influx of immune cells into adipose tissue therefore makes obesity a frequent source of prolonged proinflammatory cytokine production [38]. Importantly, in the context of the nervous system, high-fat diets—particularly those rich in saturated fatty acids (SFA)—can activate the TLR4–IKKβ/NF-κB pathway in the hypothalamic arcuate nucleus, triggering neuroinflammation and impairing leptin and insulin signaling [39].

Neural inflammation, or neuroinflammation, is a distinguishing feature of various brain disorders, dysfunctions, and diseases. These conditions can be associated with peripheral metabolic dyshomeostasis resulting from the consumption of unhealthy foods or from dietary patterns. Fatty acids play a dual role in this context: they are components of high-fat diets and, as signaling molecules, they participate in inflammatory processes. Studies have shown an association between lower intake of polyunsaturated fatty acids and high intake of cholesterol and SFA with an increased risk of cognitive impairment and dementia [40].

HFD promotes the development of neuroinflammation, most often through increased expression of proinflammatory cytokines (such Tumor Necrosis Factor α (TNF-α)) or inducible nitric oxide synthase), and it also increases activation of the mTOR (mammalian target of rapamycin) pathway, which is implicated in the development of neurological and metabolic diseases. This diet influences the activity of AMP-activated protein kinase (AMPK), which acts as a “cellular energy sensor” activated in response to decreased intracellular energy availability. Data also indicate that HFD may ultimately alter synaptic synthesis of SNAP-25 (synaptosome-associated protein 25), leading to impaired synaptic plasticity [40]. Another study showed that HFD promotes local brain inflammation. Neuroinflammatory parameters in mouse brains were examined, specifically the proinflammatory markers interleukin 6 (IL-6) and monocyte chemoattractant protein-1 (MCP-1, a mediator of inflammation). A worsening of local neuroinflammation induced by HFD during acute brain ischemia was observed [34]. Leptin is a hormone secreted primarily by adipose tissue, and its circulating levels are directly proportional to fat mass. Consequently, a high-fat diet (HFD), by promoting adipose tissue accumulation, leads to increased leptin concentrations. Leptin is widely recognized as a key regulator of energy balance [41]. An additional source of leptin is the stomach, where its production is upregulated. Locally secreted leptin modulates the composition of the microbiota and contributes to pathological processes within the gastric mucosa [42].

## 5. Effects of a High-Fat Diet on the Central Nervous System

There are many articles saying that HFD negatively affects the central nervous system, including regions such as the hippocampus, hypothalamus, and microglia [43,44,45].

### 5.1. Hippocampus

The hippocampus plays a key role in memory function, cognition, and navigation. It serves as a cornerstone for understanding neural coding and inter-regional communication, i.e., how memory-directed behavior arises from the integration of different brain areas [43]. It is believed that the hippocampus is particularly sensitive to changes in dietary energy intake [44]. An analysis of the expression of 15 genes related to glucose metabolism in the rat hippocampus showed that HFD reduces the expression of GLUT3 (glucose transporter 3), IRS2 (insulin receptor substrate 2), and IDE (insulin-degrading enzyme) in males. These results suggest that a high-fat diet affects cognitive functions and brain glucose metabolism in a sex-dependent manner, with males being more susceptible to its negative effects [45]. Another study found that rats on an HFD exhibited glucose intolerance compared to a control group, with blood glucose levels remaining elevated throughout the test; additionally, females showed impaired insulin sensitivity [46].

**Figure 1:**
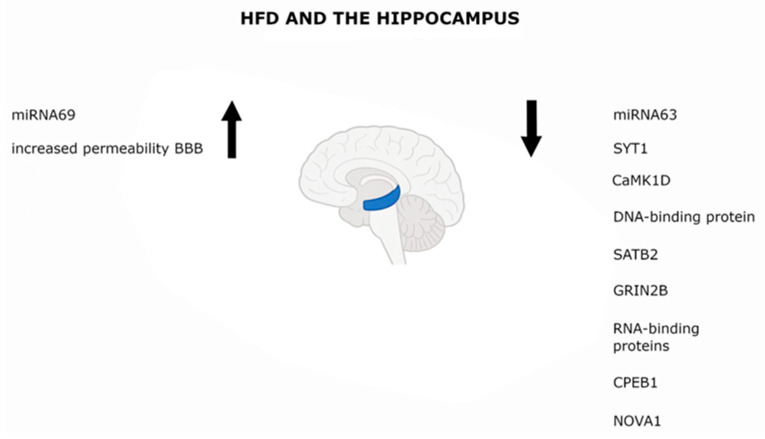
High-fat diet and the hippocampus. Upward arrows (↑) indicate an increase in the measured parameter, whereas downward arrows (↓) indicate a decrease, the hippocampus area is marked in blue.

Numerous studies have demonstrated detrimental effects of HFD on this brain region [47,48,49]. The hippocampus, a key network hub responsible for coordinating many cognitive processes, continues to develop throughout maturation, making it particularly vulnerable to environmental influences [47]. The use of HFD during critical stages of central nervous system development, such as childhood, leads to deterioration of cognitive and memory functions in both humans and animals, disrupting the function and connections among brain structures involved in emotional memory, among other things [48]. In vivo animal studies have shown that those fed an HFD exhibit impairments in hippocampus-dependent learning and memory [50]. Even a four-day exposure of rats to increased dietary fat resulted in reduced learning capacity and decreased interoceptive sensitivity (i.e., sensitivity to internal bodily cues) [49]. In a study evaluating the effect of an HFD enriched with SFA on neurogenesis in rats, reductions in hippocampal brain-derived neurotrophic factor (BDNF) levels were observed, along with increased malondialdehyde (MDA) levels and a decreased number of newly generated cells in the dentate gyrus [51]. HFD may also contribute to increased leptin secretion by enhancing STAT3 signaling in astrocytes (GFAP+ cells) in the CA1/CA3 regions of the hippocampus. A significant role of CD8+ T lymphocytes in the development of diet-induced memory deficits in aged rats has also been demonstrated [52]. T cells, as part of the immune system, are responsible for defense and antigen-specific responses. In the brain, T cells can influence the neuroimmunological environment both directly, by secreting cytokines, and indirectly, by interacting with resident microglia. In a 3-day study on young and old rats fed HFD, it was shown that a reduction in peripheral CD8+ T cells influenced hippocampal cytokine levels while simultaneously reducing neurogenesis in this region. This resulted in changes in the pre- and postsynaptic structures of the hippocampus and amygdala, which were associated with memory impairments in older male rats [53]. Another study demonstrated poorer neuronal plasticity and increased BBB permeability in the hippocampus of HFD-fed animals, as well as impaired place recognition memory [54]. These changes also affected the hippocampal miRNA expression profile in a well-established experimental model of HFD-induced metabolic disease. In the hippocampus of mice on HFD, increased expression of miRNA69 and decreased expression of miRNA63 were observed. Additionally, decreased expression of synaptotagmin 1 (SYT1), calcium/calmodulin-dependent protein kinase I delta (CaMK1D), special AT-rich sequence-binding protein 2 (SATB2), the 2B subunit of the NMDA receptor (GRIN2B), and RNA-binding proteins including cytoplasmic polyadenylation element-binding protein 1 (CPEB1) and neuro-oncological ventral antigen 1 (NOVA1) were found [55] (Figure 1).

### 5.2. Hypothalamus

The hypothalamus is a brain area responsible for regulating the body’s energy homeostasis and body weight [56]. Inflammation developing in the hypothalamus as a result of high-fat diets disrupts its proper function and affects feeding behavior and the balance between energy intake and expenditure [57]. In addition, the hypothalamus is involved in controlling hunger, thirst, stress, sleep, and the hypothalamic–pituitary axis functions [58]. Many studies have reported a strong association between dietary fat content and hypothalamic inflammation [59,60,61].

Ongoing hypothalamic inflammation is often observed in experimental animal models of HFD [62]. It is mainly triggered by an excess of saturated fatty acids, which reach brain tissue primarily via the median eminence, an area of the brain where the blood vessels have a fenestrated endothelium and are not protected by the BBB. The inflammatory process in the hypothalamus begins as early as a few hours or days after consumption of a high-fat meal [63]. It has been shown that HFD intake leads to activation of inflammatory signaling pathways such as nuclear factor κB (NF-κB) and c-Jun N-terminal kinase (JNK), which is associated with increased secretion of proinflammatory cytokines and interleukins. This process is initiated by glial cells present in the brain, mainly microglia and astrocytes, which respond to the incoming fatty acids [60]. In another study, after 3 days of feeding mice (both males and females) with a high-fat diet, hypothalamic levels of proinflammatory cytokines (interleukin 1β, IL-6, interleukin 18, TNF-α, interferon-γ), anti-inflammatory cytokines (IL-10, IL-13, TGF-β), and markers of astrogliosis (GFAP) and microgliosis (IBA1, CD68, EMR1) were determined. In males, an increase in the expression of IL-13, IL-18, IFN-γ, CD68, and EMR1 was observed, along with a slight decrease in IL-10 levels. In females, however, elevated expression of IL-6 and IBA1 was noted, together with reduced IL-13 expression. These results suggest that HFD may induce hypothalamic inflammation in a sex-dependent manner. It was also shown that in female mice fed a high-fat diet, after 3 days an increase in the expression of IL-6 and IBA1 and a decrease in IL-13 were observed, whereas after 24 weeks, elevated transcripts of IFN-γ and IL-18 were found, along with activation of astrocytes and microglia [57].

**Figure 2:**
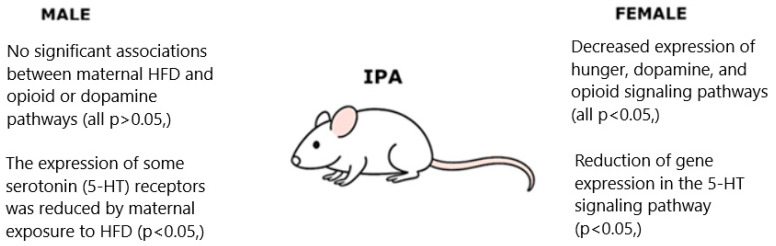
Impact of Maternal High-Fat Diet (HFD) on Serotonin, Dopamine, and Opioid Signaling Pathways in Offspring in a Sex-Dependent Manner.

Studies on mice have shown that after 2 months of HFD use, glucose concentration in the hypothalamus increased by an average of 20–30%, and the level of amino acids and neurotransmitters such as GABA and glutamate was increased by approximately 15–25%, indicating a significant change in the metabolic activity of this brain structure [63,64]. A high-fat diet may also directly stimulate orexigenic neuropeptide signaling pathways in the hypothalamus (including enkephalin, galanin, orexin, and melanin-concentrating hormone), leading to the development of an inflammatory state. Additionally, inflammatory mediators, by inducing protein expression, increase the levels and activity of these neuropeptides both in the hypothalamus and in the network of gonadotropin-releasing hormone (GnRH) neurons. Interestingly, it has been shown that direct administration of these peptides into the hypothalamus can further stimulate excessive intake of a fat-rich diet [65]. The maternal use of HFD also has a significant impact on the offspring. A maternal diet rich in fats during lactation disrupts neurotrophic development and early postnatal hypothalamic neurogenesis, regardless of the offspring’s sex. Functional pathway analysis (IPA) focused on mechanisms regulating energy balance revealed significant, sex-dependent alterations. In female offspring exposed to maternal HFD, a marked reduction in the activity of pathways related to hunger, dopaminergic signaling, and opioid signaling was observed. Modeling results suggest that the decreased expression of 5-HT receptors in the hypothalamus may result from a reduced number of POMC neurons caused by prenatal exposure to maternal HFD (Figure 2) [66].

### 5.3. Other Brain Structures Sensitive to HFD

The ventral tegmental area (VTA) and the nucleus accumbens (NAc) form the core of the “reward system”, whose plasticity is susceptible to high-fat diets. In rodent models, deficits in reward system reactivity and a compulsive overeating component have been demonstrated, associated with D2 dopamine receptor (DRD2) signaling [67]. After 6 weeks of HFD, dopamine reuptake in the NAc slows down independently of body weight [68], and impaired insulin modulation of DA transport in striatal terminals confirms the presynaptic nature of these changes [69]. Acute exposure to HFD can activate the reward system via the orexin system (an OX1R antagonist abolishes the effect), while exposure during adolescence enhances amphetamine sensitization [70,71].

The substantia nigra pars compacta (SNc) provides dopaminergic innervation essential for motor functions. Evidence suggests that HFD may impair dopaminergic neuron function and increase their vulnerability to damage: motor performance deterioration and involvement of JNK in the midbrain have been described [72], as well as enhanced dopaminergic degeneration induced by MPTP in animals with HFD/obesity [73,74].

The prefrontal cortex (PFC) exerts executive control and inhibition of reward responses. A bidirectional relationship exists between obesity/type of diet and PFC structure and function, which translates into deficits in eating self-regulation and executive functions [75].

In the case of the amygdala, HFD promotes increased proinflammatory signaling within this region. In a mouse model, IL-1β levels in the amygdala positively correlated with the severity of anxiety-like behaviors, linking the emotional–motivational component with an inflammatory background [76].

The cerebellum shows distinct, region-specific susceptibility to the effects of a high-fat diet. In a classical 16-week mouse model, inflammation involving astrogliosis, microgliosis, and increased cytokines was found to be significantly stronger in the cerebellum than in the cortex, highlighting the differential vulnerability of specific CNS regions to dietary factors [77].

### 5.4. Microglia

High-fat feeding contributes to the development of inflammation and activation of hypothalamic microglia. Additionally, inflammation induced by HFD contributes to cognitive impairments through activation of microglia in the hippocampus. Microglia, which are the brain’s equivalent of macrophages, actively monitor the local microenvironment and support tissue homeostasis under physiological conditions. In this role, they participate in synaptic remodeling, neurogenesis, removal of redundant neurons, and clearance of cellular debris [78]. These are the immune cells of the brain, defending it by producing proinflammatory compounds: TNF-α, IL-1β, IL-6, chemokine (C–C motif) ligand 2 (CCL2), superoxide radicals, reactive oxygen species (ROS), nitric oxide (NO), and matrix metalloproteinase 12 (MMP12) [78]. HFD induces a rapid but transient increase in the mRNA expression of uncoupling protein 2 (UCP2) in the mitochondria of microglial cells, leading to microglial activation and neuroinflammation. An increase in levels of proinflammatory cytokines IL-1β, IL-6, and TNF-α, as well as in Cx3cr1 mRNA (a marker of activated microglia), was observed after only three days of HFD. Additionally, hypothalamic inflammation induced by HFD co-occurred with microglial activation in this area, manifested as rapid changes in microglial cell morphology [79]. Studies of hypothalamic microglial phenotype changes in mice depending on the duration of HFD (3 days vs. 8 weeks) showed an increase in Iba1+ myeloid cells and activation of GFAP+ astrocytes in the hypothalamus after 8 weeks. The myeloid cell response was limited to the resident microglia and did not result from infiltrating cells. Furthermore, hypothalamic inflammation was confirmed after 3 days of HFD. Human studies have noted that microglial phenotypic changes were observable not only in individuals with BMI (body mass index) > 30, but that the degree of microglial changes correlated significantly with BMI. These findings confirm that microglia respond to high-fat diets in an area-specific manner in both rodents and humans [80]. Recent reports from mouse studies indicate the involvement of HFD in traumatic brain injury (TBI). A fat-rich diet may promote the development of altered microglial states in the brain. Chronic HFD, but not TBI itself, resulted in increased resting blood glucose levels and also affected the level of MCP-1 in serum and the homeostasis of adipose macrophages. It is likely that HFD selectively exacerbated the cognitive deficits induced by TBI, while amplifying transcriptomic changes in brain microglia that point to activation of inflammatory pathways [81].

Unlike short-term HFD exposure, chronic diet-induced obesity leads to long-lasting changes in hippocampal microglia: it reduces the number of dendritic spines in excitatory synapses (in two populations of dorsal hippocampal neurons: granule cells of the dentate gyrus and pyramidal cells of the CA1 region) and causes memory impairments. Importantly, it has been shown that these cognitive deficits can be partially reversed by suppressing microglial activity, which limits their phagocytic function [82]. A recent study found that HFD in adolescence induced a sex-specific transcriptional response in cortical microglia. HFD increased the expression of Itgam (encoding CD11b), Ikbkb (encoding IKKβ, a key kinase in NF-κB signaling), and Apoe (apolipoprotein E) in cortical microglia of both sexes, whereas expression of adrenergic receptor genes (Adrb1, Adra2a) changed in response to stress exposure independent of diet [83].

### 5.5. Astrocytes

Astrocytes are a key component of the brain’s immunometabolic axis and respond to HFD by rapidly transitioning into a reactive state, particularly in the hypothalamus. In rodent models, HFD activates the IKKβ/NF-κB pathway in astrocytes, the presence of which is required for the development of diet-induced obesity (DIO) and local neuroinflammation; genetic or pharmacological attenuation of this axis reduces body weight gain and cytokine expression [84,85]. HFD and SFA signal, among others, through TLR4–MyD88, enhancing the IKKβ/NF-κB/JNK cascade and the secretion of TNF-α/IL-6, which promotes leptin and insulin resistance of arcuate nucleus neurons [86,87]. Furthermore, astrocyte-specific deletion of the leptin receptor (astrocyte-specific leptin receptor knockout) impairs pSTAT3 phosphorylation in the arcuate nucleus and enhances DIO under HFD, indicating a direct role of astrocytes in leptin signal transduction and energy balance control [88]. The latest mechanistic data show that SFA activate the salvage pathway for NAD^+^ in astrocytes (NAMPT–NAD^+^–CD38); inhibition of CD38 suppresses hypothalamic inflammation and alleviates obesity in HFD-fed mice, establishing the NAMPT–NAD^+^–CD38 axis as an important regulator of astrocyte reactivity under fat excess [89]. Reactive astrocytes have been shown to modulate the excitability and switching of POMC/AgRP circuits, contributing to the DIO phenotype and representing a potential therapeutic target [87].

### 5.6. Neurons

HFD disrupts metabolic signaling in arcuate nucleus neurons, promoting resistance to leptin and insulin and deregulation of energy homeostasis; mechanistically, this is linked, among others, to activation of the IKKβ/NF-κB pathway in hypothalamic neurons [85]. Both short- and long-term HFD lead to remodeling of circuits—reductions in AgRP projections to the paraventricular nucleus of the hypothalamus and changes in the excitability of AgRP/POMC neurons have been demonstrated, functionally translating into dysregulated appetite [90]. These changes are accompanied by features of damage/regeneration within the hypothalamus (gliosis), also observed in humans [39].

In addition, HFD impairs synaptic plasticity and neurotrophy in the hippocampus (reduced BDNF, weakened neurogenesis) and leads to memory deficits in animal models and in humans [50,91]. These changes are consistent with dysregulation of the hippocampal cholinergic system observed under exposure to a “Western” diet.

### 5.7. Dopaminergic System

Diets high in fat may also impair the brain’s reward system. Chronic HFD exposure promotes changes in the dopaminergic pathway, which plays a key role in motivation and emotional/contextual behaviors. It leads to increased dopamine (DA) release associated with adaptations and hypoactivity of the reward system [92]. In rodent studies, HFD caused behavioral dysfunction and altered sleep patterns linked to the dopaminergic system: reduced vigilance, prolonged REM sleep, and anxiety-like symptoms, memory impairments, anhedonia-like behavior (reduced pleasure response), and hyperactivity—symptoms reminiscent of ADHD [92]. HFD can significantly decrease the number of DA-producing neurons in the substantia nigra by affecting tyrosine hydroxylase (TH) levels, the rate-limiting enzyme in DA synthesis. In mice fed HFD, a reduced percentage of TH-positive cells was observed in the nigrostriatal pathway. Concurrently, there was a marked increase in GFAP-positive cells in the substantia nigra and striatum, indicating enhanced neuroinflammation in the nigrostriatal DA system (with an increase in microglia and astrocytes) [93]. Another experiment examined the effect of HFD during adolescence on the functionality of the mesolimbic dopamine system (the ventral tegmental area (VTA) to nucleus accumbens (NAc) pathway) using amphetamine sensitization. This pathway consists of dopaminergic neurons in the VTA projecting to limbic areas, mainly the NAc [71]. A study on male rats showed that a high-fat diet given during adolescence (pHFD) enhanced locomotor sensitization to a single amphetamine injection. The researchers linked this effect to increased adaptations in the mesolimbic dopaminergic pathway, including a larger number of active DA neurons in the VTA, increased TH expression, enhanced DA release, and increased expression of D2 receptors and c-Fos in the NAc [71]. HFD was also shown to affect mesolimbic dopamine function: mice that consumed a higher-fat diet exhibited reduced DA release—reflected in fewer D1 and D2 receptors and dopamine transporters (DAT) [94]. These findings suggest an association between HFD and the dopaminergic system, which in turn may indicate alterations in reward mechanisms and regulation of food intake [71].

### 5.8. Cholinergic System

The cholinergic system, formed mainly by basal forebrain cholinergic neurons (BFCN; including the nucleus basalis of Meynert), underlies attention and memory through its projections to the cortex and hippocampus. In Alzheimer’s disease (AD), there is profound, selective degeneration of BFCN, which correlates with cognitive deficits and constitutes the basis of the “cholinergic hypothesis”, as well as the use of acetylcholinesterase inhibitors as symptomatic treatment [95,96,97]. Within the nutritional framework, growing evidence suggests that Western/high-fat diets can dysregulate hippocampal cholinergic signaling and impair memory in animal models, most likely through interactions with a neuroinflammatory background and other neurotransmitter systems, consistently linking diet quality with the vulnerability of cognitive circuits [91].

### 5.9. Influence of Maternal HFD on Offspring

Maternal high-fat nutrition has important consequences for offspring development. Offspring of mothers fed a high-fat diet experience altered neurological development both prenatally and postnatally [98]. Chronic maternal HFD can lead to various neurodevelopmental disorders in the progeny, such as schizophrenia, autism, depression, anxiety, or ADHD [54]. For example, a saturated-fat-rich maternal diet can reduce hippocampal volume in mouse offspring. Prenatal and postnatal exposure to maternal HFD has been shown to increase the total brain volume of the offspring, with enlargement of the medial amygdala and basal ganglia structures [99]. Another study found that maternal obesity (following HFD) adversely affected dendritic branching in newly generated hippocampal neurons of young male offspring, resulting in fewer dendritic branches and shortened total dendrite length. Additionally, the offspring of HFD-fed mothers exhibited increased lipid peroxidation in the hippocampus and decreased BDNF levels [100]. Maternal HFD also impacts offspring glial cells (oligodendrocytes, astrocytes, and microglia). In male offspring of HFD mothers, researchers observed a reduced number of oligodendrocytes and disrupted maturation, which may lead to insufficient myelination (hypomyelination) [101,102]. Significant elevations were noted in serum concentrations of proinflammatory cytokines in offspring, including interleukins IL-1β, IL-2, IL-5, IL-6, IL-10, IL-12, IL-13, IL-17, as well as interferon-gamma (IFN-γ) and TNF-α [99]. It has been demonstrated that maternal HFD increases fetal susceptibility to weight gain and induces inflammatory changes in the central nervous system of the offspring. These offspring showed reduced density of TH-positive fibers (dopaminergic projections) and decreased numbers of D1-positive and D2-positive dopamine receptor-expressing cells [102]. Mouse studies have also shown that maternal HFD affects placental function. Linoleic acid (a common fatty acid in HFD) can increase placental permeability by downregulating adhesion proteins in the placental labyrinth layer, thereby facilitating maternal–fetal transfer of substances [103]. In a rat study, adult offspring of HFD-fed mothers exhibited impaired cognitive performance. These animals had reduced hippocampal expression of the insulin receptor (Insr), the leptin receptor (Lepr), and glucose transporter 1 (Slc2a1), which persisted into adulthood [104]. This is likely linked to changes in hypothalamic circuits controlling food intake. Indeed, HFD has been associated with altered expression of neuropeptide Y, agouti-related protein, and proopiomelanocortin in the hypothalamus, suggesting that orexigenic pathways are activated by maternal HFD [105]. Another study found that in the cortex of offspring from HFD mothers, microglia were located closer to blood vessels and formed more vascular connections. This may indicate a role for microglia in cortical hypervascularization in the offspring [106]. Moreover, markers of cellular stress were observed in these offspring, along with decreased lipid bodies and reduced expression of Cyp7a1 (involved in cytochrome P450 function and lipid metabolism). Changes in the expression of the tumor-suppressor gene Btg2 and immune response genes (Csprs, Igtp) suggest potential alterations in cell cycle regulation and immune mechanisms in the cortex of offspring from HFD-exposed mothers (Table 1) [106].

## 6. High-Fat Diet and Neurodegenerative Diseases

The ketogenic diet, as a specific form of HFD, is considered a strategy both for alleviating symptoms and influencing the course of various neurodegenerative diseases (Figure 1). Recent studies indicate that only strict, long-term adherence to KD can effect a sustained metabolic shift from glucose utilization to nutritional ketosis, which is a key condition for its clinical efficacy [107]. KD exhibits neuroprotective and antioxidant properties: it supports mitochondrial function, modulates neurotransmitter activity, and limits neuroinflammation and oxidative stress [108].

Neurogenesis is a multistep process in which stem cells located in the hippocampus proliferate and differentiate into new neurons and other brain cells. Diet is one of many factors that can influence this process [109]. Neurodegenerative diseases are a major cause of disability in humans, characterized by progressive loss of especially vulnerable neurons, distinguishing them from neuronal damage caused by metabolic disturbances or toxins [110]. Recent and growing evidence from both human and animal studies suggests that KD has beneficial effects on the course of neurodegenerative diseases. This includes regulation of central and peripheral metabolism, improved mitochondrial function, and reduced inflammation [111].

### 6.1. Alzheimer’s Disease

Alzheimer’s disease (AD) is a complex neurodegenerative disorder with heterogeneous causes. It manifests as progressively worsening cognitive abilities, such as disorientation and memory loss, which impair daily functioning. Changes in behavior and personality are also common. In the brains of AD patients, elevated levels of β-amyloid (Aβ) aggregate into extracellular amyloid plaques, and hyperphosphorylated tau protein (tau-p) forms intracellular neurofibrillary tangles (NFTs). Normally, a high Aβ level in cerebrospinal fluid (CSF) and plasma, along with low deposition in brain tissue, is considered physiologically normal [112]. In Alzheimer’s disease, CSF Aβ42 is typically reduced in parallel with increased cerebral deposition, whereas in physiological aging CSF Aβ42 is relatively higher and no significant brain deposition is present.

**Figure 3:**
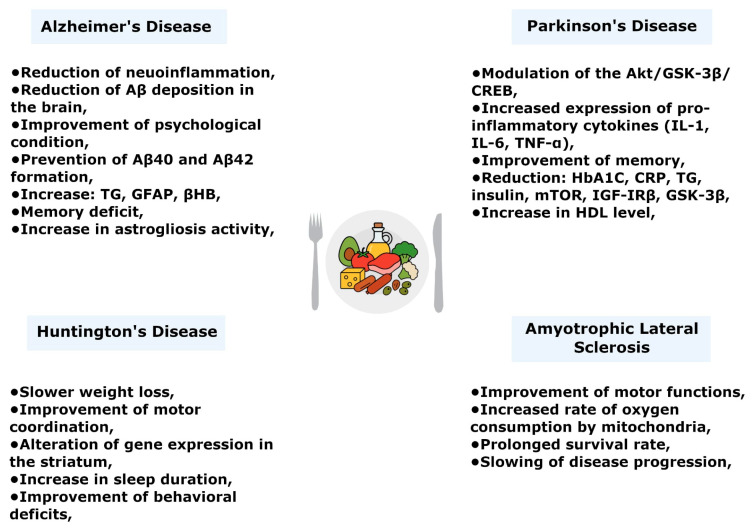
Effects of the ketogenic diet in neurodegenerative diseases.

Most studies report a positive effect of KD on AD progression. The anti-inflammatory mechanism of KD includes, among others, inhibition of microglia and astrocyte activation and reduction in the expression of proinflammatory cytokines such as IL-1β, IL-6, and TNF-α. Additionally, KD increases brain-derived neurotrophic factor (BDNF), which promotes improved synaptic maintenance and reduced amyloid-β deposition [108,111]. In one study of patients diagnosed with AD, significant improvement in mental state was observed after KD intervention. However, a noteworthy increase in serum triglycerides (TG) and LDL cholesterol was also found, which could raise the risk of cardiovascular and cerebrovascular diseases [113]. Animal studies support these findings. Mice on KD showed a reduction in Aβ burden in brain homogenates and lower levels of both Aβ and tau protein (Figure 3). These changes were associated with clear improvements in cognitive function after only 40 days on the diet. The reduction in amyloid burden helped block neurotoxicity, preventing synaptic dysfunction and neuron loss. Additionally, astrocytes metabolize KB to provide energy to neurons, emphasizing the role of genes such as GLUT1 and MCT1 in the transport of glucose and KB [108]. In mouse models, ketone bodies themselves exhibited therapeutic properties: they prevented formation of toxic Aβ40 and Aβ42 plaques. KD-fed mice lost weight, showed increased βHB levels, and had significantly lower levels of soluble Aβ40 and Aβ42—reducing total brain Aβ by about 25% (Figure 3) [114]. Another clinical study examined MCT oil supplementation in AD patients. Subjects around 70 years old received a single dose of MCT oil—there were no immediate changes in cognitive test scores. However, after 12 weeks, participants showed improvements on the Trail Making Test, the Wechsler Adult Intelligence Scale (WAIS-III), and the Wechsler Memory Scale. The authors concluded that maintaining constant nutritional ketosis is not necessary to achieve cognitive benefits from elevated ketone levels [31]. On the other hand, some studies suggest negative effects of HFD or KD in AD models. A study by Stephen L. et al. found that an HFD regimen worsened animal performance in anxiety tests, spatial memory tasks, and quality-of-life measures. HFD also increased brain levels of GFAP (glial fibrillary acidic protein, an astrocyte marker). Additionally, adverse effects were noted in the sexual behavior of subjects, suggesting that sex differences in study design may affect outcomes [115]. Another mouse study reported that HFD caused deficits in spatial memory in the Morris water maze, impaired spatial learning, more severe astroglial activation, and more β-amyloid plaques in female mice. In both sexes, HFD induced a prediabetic phenotype (Figure 3) [116].

In summary, many studies report a beneficial effect of KD in AD, but there is conflicting evidence and a need for more research, especially in humans. The current data are not uniformly conclusive.

### 6.2. Parkinson’s Disease

Parkinson’s disease (PD) is the second most common neurodegenerative disease. It is characterized pathologically by Lewy bodies and Lewy neurites, along with loss of neurons in the substantia nigra (SN) and other brain regions. Progressive motor deficits lead to increasing disability, limiting daily activities and reducing quality of life [117]. PD involves infiltration of peripheral immune cells and activation of microglia and astrocytes, indicating ongoing inflammation in the nervous system [118].

Recent studies have explored KD in the context of PD. In a rat PD model, KD had a positive effect on motor function, including neuroprotection against the toxic effects of 6-hydroxydopamine (6-OHDA). In vitro, β-hydroxybutyrate demonstrated neuroprotective effects against 1-methyl-4-phenyl-1,2,3,6-tetrahydropyridine (MPTP) toxicity in dopaminergic neurons [108]. The anti-inflammatory effect of KD may be linked to modulation of the Akt/GSK-3β/CREB signaling pathway via histone acetylation at the promoter of the mGluR5 receptor in a rat model of PD. KD increased expression of proinflammatory cytokines (IL-1, IL-6, TNF-α) but also reduced the number of microglia and dopaminergic neuronal degeneration (Figure 3). An important consideration is that ketosis induced by KD takes time (several days) to stabilize. After PD-related damage has occurred, it may be too late to introduce KD as a neuroprotective intervention [119]. Another study in human PD patients showed that reducing carbohydrates and inducing ketosis improved executive function and memory [120]. A 68-year-old woman with early-stage PD (Hoehn and Yahr stage I) followed a KD (70% fat, 25% protein, 5% carbs) for 24 weeks. Significant improvements were observed in all analyzed health biomarkers: decreases in HbA1c (Glycated hemoglobin), C-reactive protein (CRP), TG, and fasting insulin levels. She also lost weight and showed reduced cardiovascular risk factors. HDL cholesterol increased, and anxiety symptom scores improved at both 12 and 24 weeks [121]. However, a more recent study demonstrates an adverse effect of the KD. The research was conducted on transgenic mice expressing human wild-type α-synuclein (α-Syn mice), in a well-established model of Parkinson’s disease. These mice exhibited metabolic dysfunction, suggesting a link between abnormal accumulation of α-synuclein and disrupted energy homeostasis. The levels of IGF-IRβ (insulin-like growth factor I receptor β), GSK-3β (glycogen synthase kinase-3β) phosphorylated at serine 9, and mTOR phosphorylated at serine 2448 were significantly lower in α-Syn-HFD mice. Both GSK-3β and mTOR regulate numerous IGF-IRβ-dependent processes that become impaired in the course of Parkinson’s disease—including apoptosis, autophagy, neuronal metabolism, protein synthesis, and synaptic plasticity (Figure 3) [122].

### 6.3. Huntington’s Disease

Huntington’s disease (HD) is a neurodegenerative disorder characterized by neuropsychiatric symptoms, involuntary choreiform movements, and progressive cognitive decline [123]. The disease is caused by a mutation in the gene on chromosome 4 that encodes huntingtin: an expanded CAG trinucleotide repeat (encoding glutamine). When the number of CAG repeats exceeds 39, HD is inevitable and typically manifests in midlife [124]. In Huntington’s disease, the earliest and most prominent damage involves medium spiny neurons (MSN) of the striatum and cortical projections (pyramidal neurons, upper motor neuron circuits), leading to dysfunction of cortico-striatal loops and an extrapyramidal phenotype [125,126].

The effects of KD have been tested in transgenic HD mouse models. In R6/2 (Line 1J) mice, which exhibit progressive weight loss (a hallmark of HD), KD (which normally causes weight loss in healthy animals) actually slowed the weight loss in these HD mice. KD did not significantly affect the overall progression of motor impairment (i.e., it neither improved nor worsened it), but male mice on KD showed improved motor coordination [127]. In another study using the BACHD mouse model (with a controlled expression of mutant human HTT with 97 CAG repeats), transcriptomic analysis (NanoString) revealed that KD induced changes in striatal gene expression. KD lengthened daytime sleep duration and reduced sleep latency. It also improved the regularity and amplitude of activity rhythms and reduced excessive daytime activity and variability in activity onset [128]. Mutant huntingtin protein (mHTT) interferes with the transcription of genes regulated by sterol regulatory elements (SRE), which are crucial for cholesterol synthesis. It has been shown that increasing cholesterol and ketone body levels in the brain, or enhancing cholesterol efflux, can restore some disrupted synaptic functions. Animals receiving KD exhibited slower weight loss and improved behavioral deficits (Figure 3) [129].

### 6.4. Amyotrophic Lateral Sclerosis (ALS)

Amyotrophic lateral sclerosis (ALS) is a fatal neurodegenerative disease of motor neurons, leading to progressive muscle weakness and paralysis. Patients with ALS experience asthenia (fatigue), and advancing muscle weakness severely impairs quality of life and the ability to perform daily activities [130]. Increasing attention has been given to the influence of diet and metabolism on ALS. Various nutrients have been studied (including vitamins, proteins, and antioxidants), but there is no conclusive evidence of their beneficial or detrimental effects [131].

One animal study found that feeding an ALS model (transgenic) mice a diet supplemented with caprylic triglyceride (a medium-chain triglyceride used as KD) significantly improved motor function compared to controls. Treated animals showed increased basal and maximal oxygen consumption rates in spinal cord mitochondria and elevated blood ketone levels. This suggests that supplemental ketones can provide an alternative energy source for cellular metabolism in ALS-affected animals [130]. It has been proven that the survival rate in individuals suffering from ALS increased with the use of the KD. In the study, the group consuming KD exhibited the slowest disease progression. A high-calorie diet helps maintain body weight in patients with ALS and may serve as a simple, inexpensive form of therapy. Additionally, the authors report that studies on mice with SOD1 mutations (Gly86MAG and Gly93ALA) showed that calorie restriction shortens lifespan, whereas a high-calorie, high-fat diet increases body weight and slows the progression of ALS (Figure 3) [131].

## 7. Conclusions

In conclusion, the high-fat diet remains a complex and controversial nutritional model that, despite some potential benefits, is strongly associated with numerous health risks extending far beyond the central nervous system. Evidence consistently highlights digestive complications, liver dysfunction, metabolic disturbances, and micronutrient deficiencies as critical adverse outcomes that must not be overlooked. Although current research has provided valuable insights, the reliance on animal models and the variability of results depending on dietary composition, duration of intervention, and individual factors such as age, sex, or developmental stage underscore the limitations in drawing definitive conclusions for the human population. Given the growing popularity of high-fat dietary approaches, especially in the context of rapid weight loss, further well-designed studies are urgently needed to clarify the balance between their risks and potential benefits in clinical practice.

## 8. Perspectives and Future Research Directions

Future research should focus on clearly identifying which types of fats and dietary patterns exert harmful versus potentially protective effects on the central nervous system. There is a need for translational studies involving human participants, utilizing modern tools such as neuroimaging, spectroscopy, and inflammatory and metabolic biomarkers. Particular attention should be given to the impact of diet on gut microbiota and its interactions with the brain (the gut–brain axis), as well as the analysis of epigenetic effects. It is also worth designing studies on possible dietary interventions in high-risk populations for the development of neurodegenerative diseases.

## Figures and Tables

**Table 1:** Effect of high-fat diet (HFD) on the metabolism of selected brain regions within the central nervous system. Upward arrows (↑) indicate an increase in the measured parameter (e.g., body weight, food intake), whereas downward arrows (↓) indicate a decrease (e.g., synaptic markers, receptor expression).

Brain Structure	Model	Sex/Species/Strain	Age (When Diet Started/at Assessment)	Effect of HFD	Ref.
Hippocampus	High-fat, refined-sugar (HFS) diet vs. low-fat complex carbohydrate (LFCC)	Female, Rat, Fisher 344	Start: 2 months old; assessed after 2 mo, 6 mo, or 2 years of diet	Impaired hippocampal-dependent memory; altered synaptic markers)	[51]
Hippocampus	High-fat diet (HFD) model of insulin resistance	Mouse (strain not specified in the main article text visible in PDF); “male mice” stated	Age not specified in the visible Methods; behavioral tests at end of diet regimen	↓ SYT1, CaMK1D, GRIN2B, SATB2, CPEB1, NOVA1	[55]
Hippocampus	Maternal HFD (32% fat)	Male offspring, Mouse, C57BL/6J	Dams: 5 wk; offspring E18, P21, P70	↓ BDNF, impaired dendritic development, ↓ spatial learning in young	[100]
Nucleus accumbens (NAc), VTA, medial prefrontal cortex	Periadolescent HFD exposure	Male, Rat, Long-Evans	Start: PND 21; assessed PND 110–120	Reduced D2 receptor binding; impaired dopamine function	[71]
Hypothalamus, microglia	HFD with Ucp2 manipulation	Male and female, Mouse	Adult	UCP2 mediates microglial inflammation and obesity	[79]
Hypothalamus, microglia	HFD (short- and long-term)	Male, Mouse, C57BL/6J	Start: 100–120 d	Increased microglial activation, altered plasticity	[80]
Substantia nigra (SN), VTA	Chronic HFD (60% kcal fat, 20 wk)	Male, Mouse, C57BL/6J	Start: ~3 wk; assessed ~26 wk	Loss of dopaminergic neurons, ↓ PPARα/β/γ, dendritic spine loss, anxiety and cognitive deficits	[93]
Prefrontal cortex (PFC)	Maternal HFD (37% kcal fat)	Both sexes, Macaque (*M. fuscata*)	Dams 3.5–9.5 yr; offspring 7.5 and 13 mo	↑ Palatable food intake; ↓ DA fiber density, ↓ D1/D2 receptors	[102]
Cortex (vascular system), microglia	Maternal HFD (60% kcal fat)	Both sexes offspring, Mouse, C57BL/6N	Dams: 6 wk; offspring P21–P85	Hypervascularization, altered microglia–vascular interactions, ↑ stereotypic behaviors	[106]
Hippocampus, amygdala	Short-term HFD (60% kcal fat, 3 d)	Male, Rat, F344 × BN F1	3 mo and 24 mo	Aged rats: memory deficits, ↑neuroinflammation; mediated by CD8+ T cells	[53]
Hippocampus (DG, CA1)	HFD (45% kcal fat, 12 wk) or HSD (34% sucrose, 18 wk)	Male, Mouse, C57BL/6J; Cx3cr1^GFP/+	Start: 8 wk; assessed after 12–18 wk	Cognitive deficits, dendritic spine loss, microglial activation and synaptic engulfment	[82]

## Abbreviations

The following abbreviations are used in this manuscript:

AD	Alzheimer’s disease
AgRP	agouti-related peptide
ALS	amyotrophic lateral sclerosis
AMPK	AMP-activated protein kinase
BDNF	brain-derived neurotrophic factor
BrAce	breath acetone
CAG	cytosine–adenine–guanine trinucleotide repeat
CD38	cyclic ADP-ribose hydrolase 1
CD68	cluster of differentiation 68
CNS	central nervous system
CPEB1	cytoplasmic polyadenylation element-binding protein 1
CRP	C-reactive protein
CSF	cerebrospinal fluid
CaMK1D	calcium/calmodulin-dependent protein kinase I delta
D1/D2	dopamine D1/D2 receptors
DA	dopamine
DAT	dopamine transporter
DIO	diet-induced obesity
EMR1	EGF-like module-containing mucin-like hormone receptor 1 (F4/80)
GFAP	glial fibrillary acidic protein
GLUT1	glucose transporter type 1
GLUT3	glucose transporter type 3
GRIN2B	glutamate ionotropic receptor NMDA type subunit 2B
HD	Huntington’s disease
HDL	high-density lipoprotein
HbA1c	glycated hemoglobin
HFD	high-fat diet
IBA1	ionized calcium-binding adapter molecule 1
IDE	insulin-degrading enzyme
IFN-γ	interferon gamma
IKKβ	inhibitor of kappa-B kinase beta
IL-1β	interleukin-1 beta
IL-6	interleukin-6
IRS2	insulin receptor substrate 2
JNK	c-Jun N-terminal kinase
KB	ketone bodies
KD	ketogenic diet
LDL	low-density lipoprotein
MCP-1	monocyte chemoattractant protein-1 (CCL2)
MCT	medium-chain triglyceride
MCT1	monocarboxylate transporter 1
MMP12	matrix metalloproteinase-12
MyD88	myeloid differentiation primary response 88
NAD+	nicotinamide adenine dinucleotide (oxidized form)
NAMPT	nicotinamide phosphoribosyl transferase
NAc	nucleus accumbens
NF-κB	nuclear factor kappa-B
NOVA1	neuro-oncological ventral antigen 1
NO	nitric oxide
PD	Parkinson’s disease
PFC	prefrontal cortex
POMC	proopiomelanocortin
ROS	reactive oxygen species
SATB2	special AT-rich sequence-binding protein 2
SFA	saturated fatty acids
SN	substantia nigra
SNc	substantia nigra pars compacta
SYT1	synaptotagmin-1
TBI	traumatic brain injury
TG	triglycerides
TH	tyrosine hydroxylase
TLR4	Toll-like receptor 4
TNF-α	tumor necrosis factor alpha
UCP2	uncoupling protein 2
UFA	unsaturated fatty acids
VTA	ventral tegmental area
mTOR	mechanistic (mammalian) target of rapamycin
βHB	β-hydroxybutyrate
